# Microplastics in Terrestrial Domestic Animals and Human Health: Implications for Food Security and Food Safety and Their Role as Sentinels

**DOI:** 10.3390/ani13040661

**Published:** 2023-02-14

**Authors:** Joana C. Prata, Patrícia Dias-Pereira

**Affiliations:** 1 TOXRUN—Toxicology Research Unit, CESPU, University Institute of Health Sciences (IUCS), 3810-193 Gandra, Portugal; 2 Food Microbiology and Technology Laboratory, Department of Aquatic Production, Instituto de Ciências Biomédicas Abel Salazar (ICBAS), University of Porto, R. Jorge de Viterbo Ferreira 228, 4050-313 Porto, Portugal; 3 Department of Pathology and Molecular Immunology, Institute for the Biomedical Sciences Abel Salazar, Porto University (ICBAS-UP), 4050-313 Porto, Portugal

**Keywords:** plastic particles, foreign bodies, microplastics in biota, pets, companion animals, animal sentinels, bioindicators, human health

## Abstract

**Simple Summary:**

Microplastics are widespread environmental contaminants comprised of plastic pieces <5 mm. Human exposure to microplastics has been confirmed by their presence in multiple tissues. Terrestrial domestic animals may be relevant to human exposure, as they are also subjected to the same environmental contaminants, possibly acting as sentinels (i.e., help estimate exposure and risk to humans) or by being an integral part of the human food chain, as in the case of livestock. This work addresses how microplastics may impact terrestrial domestic animals, leading to consequences for food security (i.e., the availability of food) and food safety (i.e., the prevention of foodborne illnesses) and how companion animals may help estimate environmental human exposure.

**Abstract:**

Terrestrial domestic animals are exposed to microplastics, therefore, contaminating the food chain, in the case of livestock, or acting as sentinels for human exposure, in the case of companion animals. The aim of this review was to address the importance of terrestrial domestic animals on human exposure to microplastics. Animal products may already show some microplastics contamination, which may occur during their lifetime, possibly also compromising productivity, and during processing, originating from equipment and packaging. Moreover, release of microplastics in animal feces (or manure) leads to the contamination of agricultural fields, with possible impacts and internalization in plants. Therefore, microplastics pose a threat to food security, compromising food productivity, and food safety, by being a foreign material found in animal products. Conversely, in urban environments, companion animals (cats and dogs) may be relevant sentinels for human exposure. While oral exposure may vary in pets compared to humans, due to indiscriminate ingestion and chewing or licking behaviors, airborne exposure is likely to be a good indicator for human exposure. Therefore, future studies should address the importance of terrestrial domestic animals for human exposure of microplastics, both in the food chain and as sentinels for environmental exposure.

## 1. Introduction

Microplastics are particles <5 mm, which can result from intentional production (primary) or fragmentation of larger plastics (secondary), and which are considered ubiquitous contaminants due to their widespread distribution, leading to environmental and dietary exposures [1]. Microplastics have been found in indoor air [2], drinking water [3], alcoholic drinks (e.g., beer), sea salt [4], and food (e.g., fish fillet) [5]. Not surprisingly, human samples were also found to contain microplastics, such as lungs [6] and placentas [7], raising concerns over the potential adverse human health effects. However, so far, no direct toxic effects have been determined after environmental human exposure, although the consequence of extreme occupational exposures (e.g., interstitial lung disease) and potential toxicity pathways (e.g., oxidative stress) have been identified [8]. Indirect effects, such as the disruption of food production, have been suggested when considering a One Health approach [9]. Most studies have been focused on the marine environment, including on aquatic animals, despite the importance of terrestrial ecosystems on human exposure. Moreover, studies conducted on the terrestrial environment generally focus on plants [10] or invertebrates, such as snails [11]. Few have been conducted on vertebrate terrestrial animals. To the best of the authors’ knowledge, only one study has collected internal tissue samples from terrestrial animal necropsies, focusing on companion animals [12]. Otherwise, microplastics have only been found in animal feces [13] or in consumer products (e.g., meat) [14]. Moreover, while few data are available for terrestrial domestic animals, which usually live close to human populations, no integration of current data has so far been conducted on its consequences to human health. Therefore, the objective of this work was to address the importance of terrestrial domestic animals to the human health assessment of microplastics, by considering impacts on food security and food safety and their potential role as sentinel species.

A literature review was prepared based on original works on microplastics in terrestrial domestic animals available in January 2023. Literature research was conducted on Web of Science, Scopus, and Google Scholar using a combination of the keywords for the contaminant (“microplastics” or “plastic particles”) with subjects (“animals”, “companion animals”, “livestock”, “cat”, “dog”, “goat”, etc.) or animal products (“meat”, “milk”, “honey”, etc.). Finally, missing works were identified in the references of the selected articles. The final database of articles included all information available at the time on microplastics in terrestrial vertebrate animals. Two main topics were identified in the literature, which were organized in the two sections of this review. The first part addresses food security and food safety, considering their impact on animal production, contamination of animal products, and indirect effects through the application of contaminated manure. The second part addresses how terrestrial domestic animals, especially cats and dogs, could represent an untapped resource to help estimate human exposure, especially to airborne microplastics.

## 2. Microplastics as a Food Security and Food Safety Threat on Livestock

Microplastics may pose a threat to food security, by reducing the efficiency of food production, and to food safety, by being a physical contaminant of food. Indeed, multiple works suggest the presence of microplastics in terrestrial animals used in food production (Table 1). At production, animals may be exposed to microplastics, which can compromise their energy balance. A microplastic concentration of 123.9 [15], 5500 [16], and 600–3500 MP kg^−1^ was found in fishmeal and 1230 MP kg^−1^ in soybean meal [17]. Meals could be used to complement the existing animal diet, as a source of protein, or used in the formulation of animal feeds, therefore exposing livestock to microplastics through the oral route. Moreover, animals could ingest plastics when foraging, such as by ingesting contaminated invertebrates [13] or plants [18]. Environmental exposure could also play a part, such as in honeybees which contact with fibers deposited on flowers’ surface [19]. Animals could even intentionally ingest plastic foreign material, especially during starvation periods. For instance, 23.4% of cattle, sheep, and goats in an abattoir in Ethiopia presented ruminal foreign bodies, with 46.1% of objects being plastic bags, which could result in lower productivity (e.g., reduced fattening rates) [20]. In chickens, exposure to 200 mg of polyethylene microplastics per kg of feed decreased the average daily weight gain and final body weight, reduced antioxidant capacity (e.g., decreased superoxide dismutase), reduced gut microbiome diversity and abundance, and induced histopathological changes, such as liver inflammation, renal glomerular hypoplasia, and irregularities in intestinal villi morphology [21]. While the tested concentration may not translate into an average worldwide exposure, evidence suggests reduced weight gain under high exposure conditions, which could compromise food security by reducing the amount of animal products available for human consumption. This is a critical issue, considering the expected world population growth forecasted for the coming decades.

Microplastics have mainly been found in the gastrointestinal tissue of domestical animals (e.g., crop, gizzard, and intestine [13,23]). So far, little research has been conducted on muscle tissue of domestic animals, which are more often consumed. The reported absence of microplastics in chicken and goat meat when ground or cut over bamboo cutting boards [25,28] suggests that contamination may be minimal or that the current limits of detection were not able to identify smaller microplastics capable of internal distribution. Indeed, smaller microplastics sizes (e.g., <150 µm) are expected to be more easily distributed to internal tissues [36]. The main route of internalization is thought to be the gastrointestinal tract, since respiratory airways may prevent larger particles (>10 µm) from reaching the alveoli. Similarly, most particles are expected to be eliminated in the feces after biliary excretion and macrophage migration. Therefore, microplastics are often found in the feces or manure of production animals [13,27], confirming exposure. Microplastics may suffer changes due to the environment in the gastrointestinal tract. For instance, rumen conditions may lead to the release of cadmium from microplastics, as suggested by in vitro studies [37], or degradation into molecules or smaller particles [38]. The release of degradation products or smaller microplastics, more capable of internalization, could lead to the contamination of animal products. Moreover, microplastics were also found contaminating other animal products, such as milk [30,31,32] and eggs [26]. Contaminants, such as pesticides and polycyclic aromatic contaminants, are often excreted by the mammary gland and found in dairy cow’s milk [39]. Therefore, future studies should explore alternative routes of excretion of microplastics in livestock, including in milk. Contamination of eggs is also not surprising due to the lipophilic nature of microplastics and their presence in human placentas [40] or the reproductive organs of female mice [41]. In this sense, forthcoming studies should broaden the diversity of tissues sampled for microplastics, especially of smaller sizes (<150 µm), also providing more detailed information on human dietary exposure.

While animal products may already be contaminated at the time of slaughter, butchering and processing may also lead to their contamination with microplastics. Food contact materials (i.e., food contact with materials during production, processing, storage, preparation, serving, and consumption) can also represent an important source of microplastic contamination in animal products. For instance, the use of plastic cutting boards represented a contamination of 0.03–1.19 MP g^−1^ in chicken meat from the United Arab Emirates and Kuwait [25] and 2.2–6.5 MP g^−1^ (0.12–1.62 mg g^−1^) in goat meat from the Middle East [28]. Meat ground or cut on a wooden board did not present contamination, washing only reduced (but did not prevent) contamination, and cooking led to the melting and recrystallization of microplastics, which could have produced degradation products with unknown effects [25,28]. Cutting boards are thought to be one of the most important routes of microbiological cross-contamination in the kitchen [42], which led to a preference for high-density polyethylene over porous wood. However, plastic does not clearly outperform wood in regard to bacterial contamination, and wear can lead to delamination and crevices, which are harder to clean [43]. Conversely, wooden cutting boards can be sterilized in the microwave, renewed mechanically (e.g., by sanding) [43], and produced from bamboo, which provides a harder and less porous surface [44]. Since the use of wooden cutting boards could prevent additional contamination with microplastics [25,28], its implication for other food safety aspects (e.g., bacterial cross-contamination) should be better explored.

Yet, other processes could introduce plastics in animal products. For instance, milk usually crosses filters (100 µm) to discard biological contaminants, which could remove larger microplastics [30] or, conversely, be a source of synthetic fibers [31]. Milk subjected to more processing generally showed a higher concentration of microplastics, with powdered milk presenting higher concentrations than milk sampled at the farm [30]. Packaging could also introduce surface contamination on animal products. Chicken meat packed in extruded polystyrene (i.e., Styrofoam) trays was washed to collect surface microplastics, which were present in concentrations of 4.0–18.7 MP kg^−1^ (2–402 µg kg^−1^) and 18–164 fibers kg^−1^ [24]. The release of microplastics has also been detected in take-out containers (3–29 MP container^−1^) [45] and when opening plastic packaging (0.46–250 MP cm^−1^) [46]. Moreover, pieces of fatty areas of cured meats (i.e., bacon, mortadella, and salami) sealed under vacuum in light-density polyethylene resealable pouches and kept refrigerated showed the presence of Raman peaks suggesting microplastic contamination after 9 to 28 days of storage [14]. This raises questions regarding the use of plastic packaging and, specifically, of synthetic casings (e.g., polyester or polyamide casings) in cured meats and sausages, besides wasting animal intestine as casings. Moreover, products with a higher surface area (e.g., ground meat) and high lipid content could be more prone to contamination. For instance, higher microplastics concentrations were found in the egg yolk (8.95 MP egg^−1^) compared with the egg white (3.40 MP egg^−1^) [26]. Plastic packaging could also lead to contamination through the migration of chemicals into the food (e.g., additives such as bisphenol A) [47] and constitutes a growing waste stream [48]. Conversely, plastic packaging helps to lessen the environmental impact of food by reducing food waste (extending the shelf-life); by being lightweight, thus reducing transportations costs; and by improving food safety [49]. Therefore, it is important to identify processes that lead to substantial contamination of animal products and consider improvements in plastic packaging design without compromising food security and food safety.

While microplastics are considered physical contaminants of food, limited information on exposure and hazard characterization may limit attempts to conduct risk assessment. Information on exposure could be achieved by sampling different animal products for microplastics. A previous study estimated a daily oral intake of 34,254 microplastics, which also included fish, crops, and water ingestion [50]. Based on the data collected in Table 1, the estimated daily intake (EDI) of microplastics from terrestrial animal products could range from 26 to 15,381 MP day^−1^ capita^−1^, following the World Health Organization model, or from 11 to 3142 MP day^−1^ capita^−1^, following European Union consumption trends from 2013 (Table 2). However, these estimates are based on limited information, with serious knowledge gaps regarding distribution in different internal tissues (e.g., liver, kidney, and fat) and in swine and cattle meat products. Moreover, these estimates are based on assessments conducted using numerous methodologies, with different detection errors and size detection limits. Future studies should focus on sampling a wide variety of products under the same methodology with a known size range (e.g., 1–5000 µm). Hazard characterization poses an even harder challenge, since there is the need to consider the heterogeneity of microplastics (e.g., polymer type, size, shape, and additives) [51], which requires considering each factor, modeling, and understanding of dose response effects, which may not necessarily be linear [52]. In vitro and in vivo assays may help understand the toxic effects of microplastics (as reviewed in [53]). While the main toxicity pathways may involve particle toxicology, oxidative stress, and inflammation, so far there is no strong evidence on the impacts of microplastics on human health [8] to motivate stricter legislative measures, which have been mainly driven by public pressure. Even in the face of supporting evidence regarding impacts on human health, mitigation measures would only focus on reducing risk to an acceptable level [54], which is unlikely to be zero and should consider the risk–benefit of such measures. For instance, the release of fragments by plastic packaging may pose a less concerning risk than the risk of microbial contamination when using other packaging materials. Therefore, future studies should focus on addressing knowledge gaps in order to allow for risk assessment of microplastics in animal products.

There are also indirect threats to food security and food safety stemming from the application of organic fertilizers contaminated with microplastics. Indeed, microplastics have been found in poultry, cattle, and swine manure (Table 1), which could result from the excretion of particles in animal feces but also from direct contamination of the manure. Sources of microplastics in manure likely originate from plastic tools and equipment (e.g., scrapers and water pipes), packaging materials (e.g., feed bags), and animal feed [27]. Moreover, plastic mulch or silage packaging could also contaminate fields and be ingested by grazing animals, which then release microplastics back to the field in their feces [29]. Long-term application of manure as organic fertilizers is thought to contribute to 43.0–75.9% of microplastics in soils [33]. Soil contamination with microplastics can increase water evaporation, increase desiccation cracking, change the bulk density, decrease water stable aggregates, and alter microbial activity [55,56]. In addition to changing soil properties, microplastics could also affect plant growth, thus affecting agricultural outputs. For instance, the exposure of wheat (*Triticum aestivum*) to microplastics decreased root and shoot lengths (i.e., decreased growth) and increased oxidative markers [57]. Microplastics can also be internalized and accumulate in plants, such as in the roots and aerial parts of rice [18], therefore creating another source of exposure. Nonetheless, manure cannot be abandoned as organic fertilizers, which would further aggravate the shortage and escalating prices of fertilizers available on the market, compromising food production and security. Moreover, alternative disposal methods would be required to eliminate manure, with worse environmental outcomes (e.g., landfilling). Globally, manure is responsible for the input of 125.3 and 24.3 Tg year^−1^ of nitrogen and phosphorus into agricultural fields, respectively, compared with 70.2 and 14.3 Tg year^−1^ from chemical fertilizers, respectively [58]. Ideally, manure should be subjected to measures to reduce the amount of contaminants present, which usually involves composting. Indeed, composting manure could promote the degradation of microplastics, especially when combined with biochar [59]. However, microplastics may also have a negative effect on composting by reducing the abundance and diversity of microbial communities, thus reducing compost quality (e.g., reducing final nitrogen content) and increasing greenhouse gas and ammonia emissions [60,61,62]. Microplastics may also compromise manure degradation by having negative effects on the microbiome of detritivore and coprovore insects [63]. Therefore, the concentration of microplastics should be reduced beforehand. Steps to reduce microplastics in compost include manual sorting, sieving, mixing with less contaminated materials (e.g., green clippings), and increasing composting temperatures (e.g., 75 °C) to promote microplastic degradation, while, conversely, mass reduction could increase synthetic particles by a factor of 2 to 5 [64]. Some of these measures could be feasibly implemented in the management of manure to reduce microplastics content, such as dilution with plant material.

## 3. The Role of Companion Animals as Sentinels for Environmental Exposure to Microplastics

**Figure 1:**
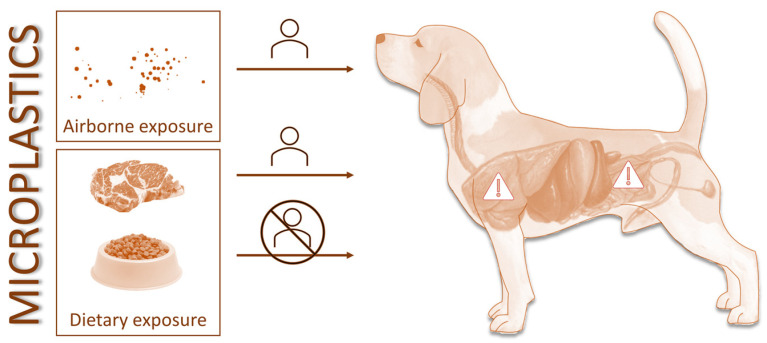
Companion animals (e.g., cats and dogs) can be good sentinels for human exposure to airborne microplastics, while dietary exposure of pets may not be shared with their family. Original artwork.

Previous works have explored the role of certain species as bioindicators (i.e., organisms which characteristics can be correlated with microplastic exposure), especially focusing on sessile aquatic species. The accumulation of microplastics in intertidal species, such as oysters (*Saccostrea forskalii*), barnacles (*Balanus amphitrite*), and periwinkle (*Littoraria* sp.), has been interpreted as resulting from the high exposure levels of the surrounding waters [66]. Moreover, mussels (e.g., *Mytilus* sp.) have been widely supported as bioindicators for monitoring microplastics in the marine environment [67]. A positive correlation has been found for the concentration and size of microplastics in waters and in the tissues of mussels (*Mytilus edulis* and *Perna viridis*) [68]. In terrestrial environments, invertebrate species, such as snails (*Eobania vermiculata*), earthworms (*Lumbricus terrestris*), decollate (*Rumina decollate*), woodlice (*Porcellio*), pill bugs (*Armadillidium*), and centipedes (*Scolopendra*), have been suggested as bioindicators [69]. However, no organisms have, so far, been suggested as sentinels for human health, especially for urban areas where higher exposure is expected. Of all domestic animals that live in proximity with human families, companion animals are the best suited as sentinels due to their shared lifestyle, similar digestive morphology (e.g., monogastric), universal distribution, and increasingly limited freedom (Figure 1).

Companion animals have been previously identified as important sentinel species. Animals can be considered as sentinels for human health since they often share the same environment and sources of food and water [70]. The monitoring of animals allows an earlier identification of environmental threats as a result of compressed lifespans, higher sensitivity, and lack of confounding behaviors (e.g., smoking) [71,72]. Companion animals, namely, cats and dogs, are especially valuable sentinels due to their role in people’s life, leading to similar exposure [73]. Thus, the use of companion animals as sentinels for human health generally results in early disease detection which allows prompt intervention. For instance, a review of 748 articles endorses the early onset of disease or mortality in companion animals exposed to polytetrafluoroethylene fumes, mercury, lead, herbicide, and asbestos in the household, preceding human cases [74]. Moreover, collection of animal samples originating from medical procedures or necropsies in companion animals are generally more easily available as they are not subjected to the same restrictions as human samples. The role of companion animals as sentinels for the environment, their higher sensitivity, and higher availability of samples could be leveraged as surrogates for estimating human exposure in the household.

Microplastics have, indeed, been found in the internal tissues of cats and dogs from Portugal subjected to necropsy, namely, in the lungs, ileum, kidney, liver, and blood clots [12]. Although this study suggests that animals are exposed in their daily environment, which is shared with pet owners, there is, so far, no data correlating exposure between pets and their families. Moreover, species-specific differences in behavior, physiology, and morphology can lead to differences in exposure. Gastrointestinal exposure may result from the contamination of the food chain, as addressed in the previous sections and supported by the presence of polyethylene terephthalate and polycarbonate in pet food [75], or from airborne deposition of particles in surfaces, plastic food bowls, food or water, and fur (i.e., during cleaning behaviors), which can then be ingested. Moreover, elongated fiber-shaped microplastics were identified in the lung tissue of domestic and fetal pigs (97 MP g^−1^) [34], which may be used as a by-product in the formulation of pet food. While pet owners may share some water and food sources with cats and dogs, there are important differences in diet composition. For instance, Portuguese companion animals are mainly fed commercial pet diets [76], which can be contaminated by the industrial processes or plastic packaging. Conversely, human dietary exposure largely varies depending on the products consumed, as addressed in previous sections. Moreover, companion animals often chew and ingest foreign materials, with plastics and rubber objects comprising 38 of 183 foreign bodies ingested by dogs and cats, the second highest category after latex teats [77]. Many pet toys are also made of plastics, likely contributing to exposure. The human digestive rate is also lower than that of carnivore animal companions (i.e., dog and cat), leading to a higher retention time of particles in the digestive system. Therefore, while companion animals may share water sources and some food with pet owners, there are likely relevant differences in oral exposure.

Exposure by inhalation can be more relevant for comparative studies, despite also requiring some species-specific considerations. First, the lower breathing height of pets can lead to a higher exposure to microplastics due to the resuspension of deposited dust. Since the complexity and surface area of nasal turbinates (i.e., convoluted nasal structures which humidify, warm, and remove particulates from the inhaled air) decrease in shorter snouts (i.e., brachycephaly) [78], cats and short-faced breeds of dogs may be more susceptible to air pollution. Indeed, indoor PM_2.5_ has been related to respiratory disease in cats (median: 38.6 µg m^−3^) but not in dogs [79]. Most particles will be deposited and cleared in the airways, with only particulate matter <10 µm (PM_10_) being able to reach the deep lungs. When larger microplastics are found in the alveoli region, they can result from rare cases where particles were able to reach the deep lung due, for instance, to their elongated morphology (e.g., fibers depending on orientation), or, more likely, from the accumulation of particles in circulation in the narrow pulmonary capillary network [80]. For dogs, only particles in circulation <8 µm are able to cross the pulmonary capillaries, leading to the accumulation of larger particles [81]. Considering the daily respiratory ventilation of 0.5 m^3^ for cats [82], the current estimation of airborne exposure is likely to be low (e.g., <8 MP day^−1^ considering an indoor concentration of 1.7–16.2 MP m^−3^ in Denmark [83]). However, most methodologies are incapable of detecting smaller airborne microplastics sizes (<10 µm), and studies have been conducted at a human breathing height. Animal exposure may be higher considering that suspended particles are generally present at higher concentrations near the soil, leading them to be more sensitive sentinels. Therefore, companion animals may be suitable sentinels for airborne microplastics <10 µm.

## 4. Recommendations and Conclusions

This work aimed at providing a novel perspective of the importance of terrestrial domestic animals on estimating human exposure to microplastics, which is required to conduct risk assessment. There are severe knowledge gaps regarding the terrestrial food chain. Terrestrial domestic animals may contact directly with microplastics in the environment or through their diet. Starvation may lead to increased ingestion of foreign materials, including plastics. Therefore, microplastics may be internalized by animals, leading to contamination at the time of collection (slaughter, milking, etc.) and possibly decrease productivity (e.g., weight gain). However, few animal products have so far been tested, especially for smaller microplastics (<150 µm) which are more prone to translocation. Processing and packaging can also increase the amount of microplastics in animal products, possibly even exceeding concentrations originally present in the product. The use of alternative non-plastic materials must be considered while guaranteeing microbiological safety. Moreover, packaging can introduce microplastics but also chemical contaminants (e.g., plastic additives) into animal products. However, plastic packaging plays an important role in food security and food safety, which must be considered in alternatives, in addition to life cycle assessments. Animal products with high lipid content or higher surface area (e.g., ground meat) may be more prone to contamination. Insufficient information is currently available to conduct risk assessment, which requires assessing human exposure (e.g., through the consumption of animal products) and hazard characterization (i.e., by understanding the human health impacts of microplastics) so that legal limits and mitigation measures can be implemented. Moreover, microplastics are excreted in animal feces and represent an indirect threat when applied as organic fertilizers to agricultural fields, contaminating plants with microplastics or reducing their productivity. Measures, such as manual sorting, composting, and dilution with plant material, may help reduce the amount of microplastics in organic fertilizers. In addition to impacts on food security and food safety, terrestrial domestic animals may also act as sentinels for human exposure. Companion animals (e.g., cats and dogs) are good sentinel species due to their proximity to human families. Companion animal necropsy samples could be leveraged to determine environmental exposure due to their higher availability. However, the interpretation of microplastics in companion animal tissues should account for species-specific behavior and morphology. Oral exposure might significantly differ despite sharing common sources (e.g., water and food scraps) with humans, as animals might chew plastics or lick their fur ingesting deposited materials. Conversely, companion animals may be a good sentinel for the inhalation of airborne microplastics <10 µm, as larger particles in the lungs likely result from circulating particles (i.e., internalized in the gut) that cannot cross pulmonary capillaries. Since animals and humans share their environment, in the home or during walks, animals may represent a sentinel species for airborne contaminants. Therefore, future studies should evaluate the potential role of companion animals as sentinels by comparing their exposure to human exposure and to environmental concentrations of airborne microplastics. The main areas that need to be addressed are (i) improving methodologies to be able to more easily determine the concentration of microplastics in multiple matrices, by using sample reduction methods (e.g., digestion followed by centrifugation) and identification methods capable of characterizing smaller particles (e.g., microscopy and micro-Raman spectroscopy); (ii) determining sources of contamination along the food chain, from farm to fork, in order to develop strategies to reduce contamination; (iii) estimating human exposure related with the consumption of animal products; (iv) assessing the suitability of companion animals as sentinels for human airborne exposure; (v) determining the human health effects of realistic concentrations of microplastics; (vi) conducting risk assessment, thus providing a preliminary foundation for risk management and risk communication. Hopefully, knowledge gaps on microplastics will be addressed by following a structured approach based on risk assessment, thus providing policy makers with the needed information to make conscious decisions.

## Figures and Tables

**Table 1:** Microplastics in products from terrestrial domestic animals.

Animal	Sample	Findings	Country	Reference
Duck	Intestine	11–49 MP intestine^−1^	Indonesia	[22]
Chicken	Crop	11 MP crop^−1^	Mexico	[13]
	Gizzard	Present	Philippines	[23]
		45.8 MP gizzard^−1^	Mexico	[13]
	Intestine	Present	Philippines	[23]
	Meat (packaged)	4.0–18.7 MP kg^−1^ 18–164 fibers kg^−1^	France	[24]
	Meat (cut)	30–1190 MP kg^−1^	United Arab Emirates and Kuwaiti	[25]
	Eggs	11.67 MP egg^−1^	China	[26]
	Feces	129,800 MP kg^−1^	Mexico	[13]
	Manure	667 MP kg^−1^ (w.w.)	China	[27]
Goat	Meat (cut)	2200–6500 MP kg^−1^ 120–1620 mg kg^−1^	Middle East	[28]
Sheep	Feces	997 MP kg^−1^	Spain	[29]
Cattle	Milk	2040–10,040 MP L^−1^	Switzerland and France	[30]
		3–11 MP L^−1^	Mexico	[31]
		40 MP L^−1^	Ecuador	[32]
	Manure	74 MP kg^−1^ (w.w.)	China	[27]
		4520 MP kg^−1^ (d.w.)	China	[33]
Pig	Lungs	180,000 MP kg^−1^	China	[34]
	Manure	902 MP kg^−1^ (w.w.)	China	[27]
		3547 MP kg^−1^ (d.w.)	China	[33]
Bee	Honey	54 and 67 MP L^−1^	Ecuador	[32]
		40–660 fibers kg^−1^ 0–38 fragments kg^−1^	Germany, France, Italy, Spain, Mexico	[19]
		10–336 fibers kg^−1^ 2–86 fragments kg^−1^	Germany	[35]

w.w., wet weight; d.w., dry weight.

**Table 2:** Estimated daily intake per capita of microplastics from terrestrial animal products.

Product	Daily Intake ^(a)^	Concentration Range ^(b)^	Estimated Daily Intake (MP day^−1^) ^(c)^
World Health Organization model			
Meat	300 g	30–1190 MP kg^−1^	9–357
Liver	100 g	n.a.	n.a.
Kidney	50 g	n.a.	n.a.
Animal fat	50 g	n.a.	n.a.
Eggs	100 g ^(d)^	12 MP egg^−1^	24
Milk	1.5 L	3–10,000 MP L^−1^	5–15,000
Europe Union trends (2013)			
Poultry meat	316 g	30–1190 MP kg^−1^	10–376
Dairy products	286 g	3–10,000 MP L^−1^	1–2766
Pig Meat	96 g	n.a.	n.a.
Bovine Meat	86 g	n.a.	n.a.

^(a)^ Conservative model used by the World Health Organization for residues in veterinary products [65]; ^(b)^ based on the data in Table 1; ^(c)^ Estimated Daily Intake (EDI) = concentration (MP g^−1^) [50]; ^(d)^ approximately 2 eggs.

## Data Availability

All data are available in the manuscript.
